# Epidemiology of premalignant and malignant upper gastrointestinal lesions among patients presenting from a rural community in Karachi, Pakistan

**DOI:** 10.12669/pjms.39.3.7379

**Published:** 2023

**Authors:** Muhammad Kamran, Abdullah Bin Khalid, Muhammad Danish Muneeb, Sohail Hussain

**Affiliations:** 1 Muhammad Kamran, FCPS. Assistant Professor, Department of Medicine, Fatima Hospital, Baqai Medical University, Karachi, Pakistan; 2 Abdullah Bin Khalid, FCPS. Assistant Professor, National Institute of Liver & GI Diseases (NILGID), Dow University of Health Sciences, Karachi, Pakistan; 3 Muhammad Danish Muneeb, FCPS. Associate Professor, Department of Surgery, Fatima Hospital, Baqai Medical University, Karachi, Pakistan; 4 Sohail Hussain, FCPS. Assistant Professor, Department of Gastroenterology, Ziauddin University, Karachi, Pakistan

**Keywords:** Upper GI Endoscopy, Gastrointestinal cancers, Rural Community, Epidemiology

## Abstract

**Objective:**

Understanding the epidemiology of upper gastrointestinal cancers in Pakistan may help in identifying important demographic risk factors for upper gastrointestinal malignancies in a particular rural population group. This will benefit in implementing tailored prevention approaches as well as effective management of health services.

**Method:**

A secondary data analysis of 1193 patients was conducted who went through diagnostic upper GI endoscopy between December 2016 to May 2019 at Fatima Hospital. The endoscopies were performed at Fatima Hospital which is the main health resource for the specifically targeted rural community. Data was analyzed using SPSS version 21.

**Results:**

The median age of patients included in the sample was 35 years (IQR=20 years). One third of all endoscopic findings were concluded as normal. The frequency of malignant upper gastrointestinal lesions was relatively higher among male and patients with age 65 years or more. The study didn’t find any significant differences in the distribution of malignancies on the basis of ethnicity. Adenocarcinoma of esophagus was the most common malignant lesion.

**Conclusion:**

The average age of patients undergoing upper gastrointestinal endoscopy among rural community of Karachi was relatively low. The burden of upper GI malignancies was significantly higher among elderly. Male patients had significantly greater burden of premalignant and malignant lesions as compared to females. No differences in the distribution of diagnostic outcomes were observed on the basis of ethnicity.

## INTRODUCTION

Pakistan is a country where burden of cancer or malignant diseases is consistently on the rise in all age groups. The commonly seen cancers among Pakistani men are lung and prostate cancers while breast and ovarian cancers are common among Pakistani females. However, the commonest or most frequent cancers in Pakistan which are equally prevalent among males as well as females are those of the oral cavity and gastrointestinal (GI) tract.^1^,^2^ Having said that, the actual incidence of various cancers in Pakistan cannot be estimated due to unavailability of a national cancer registry or lack of national surveillance system.^1^

Consequently, there is dearth of local evidence regarding various possible risk factors associated with upper GI cancers among Pakistani population. Literature reports that a high prevalence of helicobacter pylori (h. pylori), excessive smoking, alcohol use, betel quid and betel nut are the major risk factors associated with chronic gastric inflammation and upper GI malignancies in Pakistan.^3^-^5^ However, besides behavioural risk factors, regional and ethnic differences within a population might be important determinants for the distribution of GI malignancies in a community.^6^-^9^ Literature reports that cancer related morbidity and mortality among populations differs not only by differences in geographical location or ethnicity, but the urban-rural differences also play an important role.^8^,^9^

A study conducted in 2012 in China compared the Age-Standardized Incidence Rates (ASRs) for gastroesophageal cancers between urban and rural communities and analyzed the incidence of disease between the year 2000-2015. The study found considerably higher burden of gastroesophageal cancers in the rural population as compared to urban population.^8^ Understanding the epidemiology of upper GI cancers in Pakistan may help in primary as well as secondary prevention. Identifying important demographic risk factors for upper GI malignancies in a rural population group may help in designing and implementing tailored screening approaches for prevention as well as effective planning and management of health services.

## METHODS

This study was a secondary data analysis of a previously conducted retrospective cross-sectional study conducted in 2019 at Fatima Hospital.^10^ It is a tertiary care teaching hospital located at M-9 Super Highway, Karachi and is affiliated with Baqai Medical University, Karachi, the biggest local health resource for the local rural community living in the vicinity.^11^ This study included a sample of 1288 patients who went through diagnostic upper GI endoscopy, either as in-patients or as a day-care service, between December 2016 to May 2019. All the upper GI endoscopies were conducted in the endoscopy room of the Department of Medicine at Fatima Hospital.

The key variables included in this study were basic demographics characteristics, clinical symptoms relevant to gastrointestinal diseases as well as biopsy results. Ethical approval for this study was granted by the Ethics Review Committee of Baqai Medical University, Karachi (Ref: BMU-EC/07-2019-03, Dated: July 19th 2019). Data was entered and analyzed using SPSS version 21. Descriptive statistics were calculated for basic demographic and health related characteristics. Chi-square test of significance was applied to identify any statistically significant differences in the demographic characteristics of patients with benign, premalignant and malignant lesion of upper GI tract. P-value of 0.05 or less was considered statistically significant.

## RESULTS

The data of 1288 patients who underwent upper GI endoscopy between December 2016 to May, 2019 was accessed and reviewed from hospital medical records. After exclusion of endoscopies performed for therapeutic reasons, we finally obtained a sample of 1193 patients who went through diagnostic upper GI endoscopy. The socio-demographic characteristics of study participants are presented in Table-I.

**Table-I:** Demographic and health related characteristics of patients examined through Upper Gastrointestinal Endoscopy (n=1193).

Variable	Frequency(n)	Percentage (%)
***Median age 35 years (IQR=20 years)***		
***Age***		
40 years and less	802	67.2
41-64years	312	26.2
65 years and above	79	6.6
***Sex***		
Male	594	49.8
Female	599	50.2
***Ethnicity***		
Balochi	25	2.1
Punjabi	17	1.4
Pashto	596	50.0
Sindhi	455	38.1
Urdu	100	8.4
***BMI***		
Underweight	427	35.8
Normal	586	49.1
Overweight	102	8.5
Obese	78	6.5
***Patient category or type***		
In-patient	192	16.1
Out-Patient	1001	83.9
***Reported GI Symptoms***		
Chronic Diarrhea	4	0.3
Dysphagia	29	2.4
Epigastric Pain	769	64.5
Heart Burn	202	16.9
Hematemesis	20	1.7
Hiccups	4	0.3
Iron Deficiency Anemia	44	3.7
Melena	5	0.4
Screening for varices (CLD)	61	5.1
Vomiting	37	3.1
Weight Loss	18	1.5

**Fig.1:**
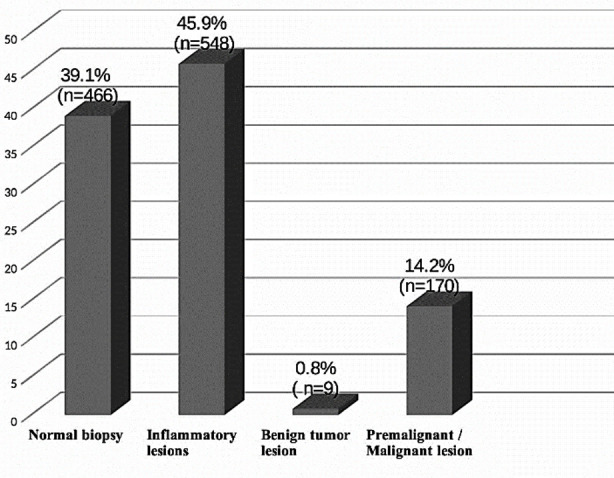
Distribution of Diagnostic Upper Gastrointestinal Endoscopy findings or lesion types according to biopsy result (n=1193).

Among the 1193 patients who went through diagnostic upper GI endoscopies, only 14.2 % (n=179) were found to have premalignant or malignant lesions, whereas only 0.8% (n= 9) were diagnosed to have a truly benign lesion. 45.9% (n=548) of the patients had upper GI inflammatory lesions while 39.1% (n=466) of all the diagnostic endoscopies were concluded as normal, since no pathology was identified on endoscopic examination. (Fig.1) The proportion of premalignant and malignant lesions was highest among elderly patients from age group 65 years and above (Table-II).

**Table-II:** Demographic and health related characteristics of patients diagnosed to have pathological findings Upper Gastrointestinal Endoscopy (n=727).

Variable	Inflammatory lesions (n=548)	Benign lesions (n=9)	Premalignant or malignant lesions (n=170)

	Frequency (%)		
***Age***			
40 years and less	365(78.3)	02(0.4)	99(21.2)
41-64 years	144(71.6)	04(20.0)	53(26.4)
65 years and above	39(65.0)	03(5.0)	18(30.0)
***Sex***			
Male	275(74.1)	5(1.3)	91(24.5)
Female	273(76.7)	4(1.1)	79(22.2)
***Ethnicity***			
Balochi	15(88.2)	0	02(11.8)
Punjabi	09(81.8)	0	02(18.2)
Pashto	265(75.5)	04(1.1)	82(23.4)
Sindhi	212(73.6)	04(1.4)	72(25.0)
Urdu	47 (86.7)	01(1.7)	20(12)
***BMI***			
Underweight	176(73.9)	3(1.3)	59(24.8)
Normal	279(75.8)	6(1.6)	83(22.6)
Overweight or Obese	93(76.9)	0	28(23.1)
***Patient category or type***			
Out-patient	459(76.1)	06(1.0)	138(22.9)
In-Patient	89(71.8)	03(2.4)	32(25.8)

The subgroup analysis of 179 patients diagnosed with benign, potentially malignant or malignant lesions showed that 79.3% (n =142) of these patients had premalignant lesions, 15.7% (n=28) had malignant lesion while 5.0% (n=9) were diagnosed to have benign tumors (Table-III).

**Table-III:** Distribution of demographic and health related characteristics among patients with Benign, Premalignant and malignant lesions of Upper Gastrointestinal Tract (n=179).

Variable	Benign lesions (n=9)	Premalignant lesions (n=142)	Malignant Lesions (n =28)	P-value

Frequency (%)			
***Age***				
40 years and less	02(2.0)	88(87.1)	11(10.9)	
41-64 years	04(7.0)	41(71.9)	12(21.1)	0.014
65 years and above	03(14.3)	13(61.9)	05(23.8)	
***Sex***				
Male	05(74.1)	70(1.3)	21(24.5)	0.04
Female	273(76.7)	4(1.1)	79(22.2)	
***Ethnicity***				
Balochi	15(88.2)	0	02(11.8)	
Punjabi	09(81.8)	0	02(18.2)	0.91
Pashto	265(75.5)	04(1.1)	82(23.4)	
Sindhi	212(73.6)	04(1.4)	72(25.0)	
Urdu	47 (86.7)	01(1.7)	20(12)	
***BMI***				
Underweight	3(4.8)	33(53.2)	26(41.9)	
Normal	6(6.7)	81(91.0)	2(2.2)	<0.001
Overweight or Obese	0	28(100.0)	0	
***Patient category***			
In-patient	06(4.2)	119(82.6)	19(13.2)
Out-Patient	03(8.6)	23(65.7)	09(25.7)

P-value of ≤ 0.05 is significant.

**Fig.2:**
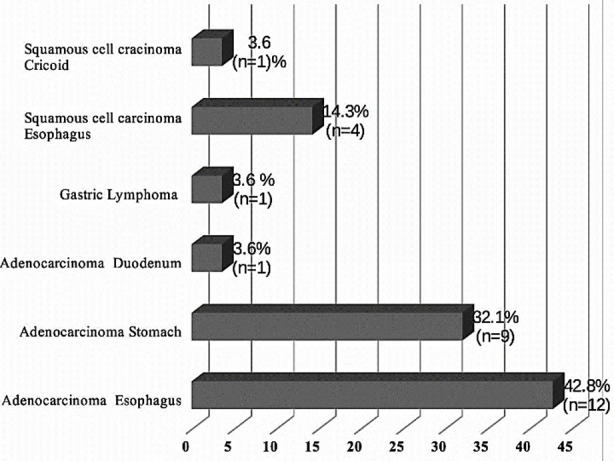
Distribution of various types of biopsy confirmed upper GI malignancy cases among patients representing a peri-urban community setting in Karachi, Pakistan (n=28).

Further analysis of biopsy confirmed upper GI malignancy cases found that adenocarcinoma of esophagus, adenocarcinoma of stomach and squamous cell carcinoma of esophagus were the most common lesions with proportions of 42.8% (n=12) and 32.1% (n=9) and 14.3% (n=4) respectively (Fig.2).

## DISCUSSION

This study is a unique contribution to evidence regarding epidemiology of upper GI tract tumors in Pakistan which include benign as well as malignant lesions. The median age of patients undergoing diagnostic endoscopy was 35 years. This indicates that the study sample comprised a considerable proportion of young patients, a finding which is different when compared to average age of endoscopy reported from previously conducted studies mainly representing urban populations.^12^,^13^ A study conducted by Farzana and colleagues at Department of Pathology, Liaquat University of Health Sciences, Jamshoro, reported that the average age of patients who went through endoscopy was 40 years.^12^ Another study conducted with a sample of 1066 patients at Dr. Ruth K.M. Pfau Civil Hospital, Karachi between January to December, 2017 reported that the mean age of patients undergoing the procedure of upper GI endoscopy was 42 years.^13^

The current study found that among all the diagnostic endoscopies, more than one third of the endoscopies failed to identify any macroscopic pathology and were concluded as normal. The distribution of endoscopic examination findings which showed highest proportion of normal examination were among the patients of age 40 years or less (not shown in results). This finding indicates that patients in young age group, i.e., 40 years or less mainly had functional upper GI symptoms resulting in normal examination findings while the frequency of upper GI disorders tend to increase after 40-55 years of age depending on the disease epidemiology as suggested by previous studies.^14^,^15^

Our findings apparently seem to support endoscopy guidelines specifically formulated for Asian countries, recommending the age limit for endoscopy as 40 years, 45 years and 50 years for countries with high, intermediate, and low prevalence of GI malignancies respectively.^16^ Interestingly, our subgroup analysis found significantly higher proportion of precancerous lesions in the younger age group i.e., patients of age 40 years or less. This finding highlights the crucial need of designing a locally tailored and more critical approach for high risk screening as per local malignancy detection rates by applying more rigorous stratification of risk factors in local context.^13^,^14^

The identification and implementation of evidence based local endoscopy criteria can also help in improving the cost-utility and related efficiency of health system.^17^ The role of advancing age and male gender in gastrointestinal epidemiology is well supported by a prospective study conducted in Northeast China as part of national cancer screening program.^18^ The study identified high risk individuals on the basis of a questionnaire based tool known as Harvard Risk Index. The endoscopy findings for the high risk group revealed a higher disease detection rate for both esophageal and gastric lesions among men as compared to women, as well as with rise in age of screening participants.^18^

Our study found considerably high percentage of benign tumors among patients of age between 41 to 64 years while a significantly greater proportion of malignant lesions was observed among patients of age 65 year or more. This finding is in contrast to previous evidence suggesting average age for gastric cancers ranging between 50 to 58 years.^7^,^16^,^18^-^20^ Nevertheless, this study found relatively higher burden of premalignant and malignant lesions among male patients as compared to females, an observation which is well supported by previously published literature.^19^,^20^ The higher burden of GI malignancies among male patients in our study can be explained by a comparatively higher burden of smoking, alcohol and other kinds of substance abuse.^16^-^18^

Our study didn’t find any significant differences in the proportion of malignant and benign lesions on the basis of ethnicity. Furthermore, the subgroup analysis also found relatively higher proportion of patients with premalignant lesions in all three BMI categories. These findings can be explained by relatively small and disproportionate sample size in certain ethnic groups and BMI categories and hence, need further exploration in the form of large studies with greater sample size.

Adenocarcinoma of esophagus was the most dominant upper GI malignancy. This finding is in contrast to a previous study conducted among rural population in Hyderabad which reported squamous cell carcinoma of esophagus as the most common type of upper GI malignancy.^21^ Such differences can possibly be explained by ethnic and geographical differences, requiring more in-depth analysis in future studies.

The study findings from this study indicate that most of the diagnostic endoscopies were performed among patients of age 41 years and less. This important observation suggests further exploration regarding related factors and appropriate interventions for the prevention of upper GI diseases in our youth including lifestyle related factors. This is important to understand the clinical significance of this emerging evidence in local context as this might be indicating changing trends in epidemiology of GI cancers and other clinical conditions with an indication for Upper GI Endoscopy.

### Limitations:

This study is a secondary data analysis of medical records; hence it has few inherent limitations. First of all, it lacks information about many basic sociodemographic characteristics such as education, socioeconomic status, occupation, and exposure to risk factors (for e.g. smoking, tobacco use and betel nut chewing) associated with upper GI malignancies. Moreover, the information collected from medical records is not sufficient enough to estimate the actual burden of upper GI cancers in the target community.

### Strengths:

Nevertheless, despite some limitations this study has obvious strengths. It offers a focused yet valuable insight into the current situation regarding epidemiology of upper GI malignancies in a rural community of Karachi, Pakistan. The study raises a valid discussion about developing a more contextualized approach while identifying appropriate candidates for upper GI endoscopy. However, the development of a contextualized evidence based eligibility criteria for upper GI endoscopy becomes more crucial in a resource limiting country like Pakistan.

## CONCLUSION

The burden of Upper GI malignancies in the rural population of Karachi was significantly higher among males in comparison to females and patients of age 65 or more. The burden of premalignant cases was higher among patients of age between 41and 64 years. Adenocarcinoma of esophagus and stomach were the most common malignant lesions.

### Authors Contribution:

**MK:** Conceived the idea of this research, developed the proposal and wrote the first draft of the manuscript. He is also responsible for the authenticity and integrity of the work.

**ABK:** Contributed as a senior author in review and revision of study methodology and plan of analysis and analyzed data.

**MDM:** Wrote the discussion in first draft of the manuscript.

**SH:** Supervised data analysis and reviewed the results and tables.
